# Jumping in simulated lunar gravity with blood flow restriction as a potential exercise countermeasure: The acute physiological effects

**DOI:** 10.1113/EP093652

**Published:** 2026-06-17

**Authors:** Patrick Swain, Filipa Santos, Nick Caplan, Cody Latue, Billie Illingworth, Nicky Sheard, Luke Hughes

**Affiliations:** ^1^ Aerospace Medicine and Rehabilitation Laboratory, School of Sport, Exercise and Rehabilitation, Faculty of Health and Life Sciences Northumbria University Newcastle upon Tyne UK; ^2^ Departamento de Física, Faculdade de Ciências Universidade de Lisboa Lisboa Portugal

**Keywords:** blood flow restriction, countermeasure, exercise, jumping, moon

## Abstract

The present study examined the cardiovascular, metabolic, neuromuscular and perceptual effects of low‐intensity jumping in simulated lunar gravity (∼20% bodyweight) with blood flow restriction (BFR). Fourteen healthy adults (24 ± 4 years; 1.81 ± 0.06 m; 75 ± 12 kg) completed an incremental jumping test in simulated lunar gravity (9.5° head‐up tilt suspension) followed by a graded exercise test to determine maximal oxygen uptake (${\dot{V}}_{O_{2}\mathrm{peak}}$). Two exercise visits (with and without BFR) were completed on separate days comprising 6 × 2 min of low‐intensity (40% ${\dot{V}}_{O_{2}\mathrm{peak}}$) continuous jumping at ∼20% bodyweight, with 1‐min passive rest periods. Blood flow restriction was applied at 60% limb occlusion pressure to the lower limbs during the exercise periods and removed during the rest periods. Jumping in simulated lunar gravity with BFR, compared to without BFR, significantly decreased muscle tissue oxygenation within the vastus lateralis (∼20%) and gastrocnemius medialis (∼5%), and increased heart rate (10–23 beats min^−1^), carbon dioxide output (2–4 mL kg^−1^ min^−1^) and minute ventilation (7–16 L min^−1^). Oxygen consumption was either unaffected or slightly elevated up to 3 mL kg^−1^ min^−1^. Blood lactate concentration was significantly greater during BFR jumping by ∼1–2 mmol L^−1^. Pre–post exercise knee extension peak force declined in both conditions to similar extents. Perceived exertion, discomfort and body instability significantly increased with BFR. Jumping in simulated lunar gravity with BFR requires little equipment and elicits similar metabolic, cardiovascular and perceptual responses to BFR aerobic exercise in terrestrial settings (e.g., BFR walking/cycling), and therefore may have value as a musculoskeletal and cardiovascular exercise countermeasure during planetary exploration missions.

## Abstract (highlights)

- **What is the central question of this study?**
  What are the acute physiological responses to bodyweight jumping in simulated lunar gravity with and without blood flow restriction (BFR)?
- **What is the main finding and its importance?**
  Low‐intensity bodyweight jumping at ∼20% bodyweight (simulated lunar gravity) with BFR, compared to without BFR, augments the acute cardiovascular, metabolic and perceptual responses to exercise. These findings provide rationale for exploring the use of bodyweight jumping with BFR as an exercise countermeasure in surface habitats during planetary exploration missions.

## 1 INTRODUCTION

Future planetary missions to the Moon (17% Earth's gravity; 0.17 *g*) and Mars (38% Earth's gravity; 0.38 *g*) require consideration towards how human health and performance can be maintained to operate nominally within extreme environmental conditions (Capri et al., 2025; Patel et al., 2020). Exposure to weightlessness (∼0 *g*), such as onboard the International Space Station (ISS), is a key contributor to ‘spaceflight‐induced deconditioning’, characterised by muscle atrophy and strength loss, loss of bone mineral density, cardiovascular deconditioning, sensorimotor impairment, compromised immune function and spaceflight‐associated neuro‐ocular syndrome (Fitts et al., 2001, 2010; LeBlanc et al., 2000; Lee et al., 2020; Norsk, 2020; Orwoll et al., 2013; Stavnichuk et al., 2020; Tays et al., 2021). Upon landing on the Moon and Mars, evidence indicates that ‘hypogravity‐induced deconditioning’ is also expected (Richter et al., 2017; Swain, et al., 2022a, 2022b).

Surface extra‐vehicular activities (EVAs) will be a fundamental activity performed during exploration missions. Mission profiles plan for crewmembers to perform up to 24 h of EVAs per week (6–8 h sessions) for scientific exploration and construction, maintenance and repair of lunar infrastructure (NASA, 2020, 2022a). The physical requirements of simulated surface EVAs and mission‐critical tasks (e.g., emergency egress) have been explored, showing that impairments in physical performance could result in task failure or delayed completion (Ade et al., 2014, 2015, 2016; Alexander et al., 2019; Ryder et al., 2019; Sutterfield et al., 2019). Future exercise countermeasure programmes, therefore, must be effective enough to prevent the physical capabilities of crewmembers falling below the physical requirements of operational tasks (NASA, 2007, 2014, 2022b).

Exercise countermeasure programmes used onboard the ISS currently schedule crewmembers up to 2.5 h per day for exercise‐related activities including hardware set‐up and stowage, aerobic (30 min) and resistive (40–60 min) exercise, and personal hygiene (Hackney et al., 2015; Petersen et al., 2016). Aerobic exercise is performed using specialised equipment including the cycle ‘Flight ERGOmeter’ (FERGO) and second‐generation combined‐operational load bearing external resistance treadmill (T2‐COLBERT). Resistive exercises (e.g., deadlifts, squats, calf raises and bench press) are performed using the Advanced Resistive Exercise Device (ARED). The ISS exercise countermeasure programme, however, does not completely protect all crewmembers from deconditioning, with notable inter‐individual variability and some astronauts showing performance‐limiting impairments (Scott et al., 2023). These are important matters of concern, as future mission scenarios expect greater constraints upon the use of exercise that has ultimately warranted the development of more effective and time‐efficient strategies for preventing multi‐system physiological deconditioning using smaller exercise hardware (Laws et al., 2020; Scott et al., 2019). Plyometrics and blood flow restriction (BFR) have independently gained recognition as unique training strategies that offer the potential to meet these new idealisms (Hughes et al., 2022; Kramer et al., 2017a; Laflamme et al., 2025).

Jumping gained interest as a potential spaceflight countermeasure owing to the remarkable success of a sledge‐jump training programme in mitigating key symptoms of disuse‐induced deconditioning including bone mass and mineral density, lean muscle mass, maximal leg strength, peak oxygen uptake and indices of sensorimotor performance, simultaneously, in participants undergoing 60 days’ bed rest (Kramer et al., 2017a, 2017b; Ritzmann et al., 2018). These findings demonstrate that it may be possible to effectively mitigate disuse‐induced physiological deconditioning with a single exercise modality and with little time commitment compared to current ISS exercise protocols, as jumping exercise session durations were approximately 10–15 min (Kramer et al., 2017b).

Jumping against one's own bodyweight within planetary habitats on the Moon and Mars could also serve as a valuable form of exercise in these unique hypogravity settings, as it requires no equipment to perform. Recent evidence from our laboratory has demonstrated that the metabolic demand of continuous jumping against one's own bodyweight in simulated lunar gravity (∼20% bodyweight) can be of a sufficient rate to facilitate oxidative phosphorylation serving as the primary energy source (Swain et al., 2025b). The metabolic cost of jumping increases linearly with jump height; however, this presents a challenge whereby surface habitat interior headroom (∼2.5 m in lunar habitat design plans) could limit the ability to exercise at desired intensities, particularly as jump heights up to ∼4 m appear possible on the Moon (Burke et al., 2022; Cavagna, 1972).

One potential solution to enhance the physiological stimulus provided during bodyweight jumping in surface habitats includes the application of BFR (Behringer and Willberg, 2019; Hackney et al., 2012; Hughes et al., 2022; Willis et al., 2019). Training with BFR involves applying a pneumatic tourniquet cuff to the proximal portion of the exercising limb(s) and inflating to a pre‐determined percentage of the individual's limb occlusion pressure (LOP) to partially and completely restrict arterial and venous blood flow, respectively. This technique can be applied during both aerobic and resistance exercise modalities. A unique feature of BFR training includes its ability to elicit training adaptations when performed at low loads (20–40% 1‐repetition maximum) or metabolic rates (<50% maximal aerobic capacity) with relatively short training sessions (∼10–20 min) (Patterson et al., 2019). Low‐intensity BFR aerobic training (e.g., BFR walking or cycling) has been observed to improve both cardiovascular fitness and muscle size and strength simultaneously, with new evidence suggesting BFR exercise may also provide an osteogenic stimulus, thus targeting key systems susceptible to disuse‐induced deconditioning (de Oliveira et al., 2016; Hughes and Centner, 2024; Jack et al., 2023). Jumping in simulated lunar gravity at metabolic rates <50% maximal aerobic capacity is certainly possible if the jump height is sufficiently low, thereby opening the door to an interesting possibility that BFR aerobic exercise could be performed in surface habitats by simply jumping on‐the‐spot (Swain et al., 2025b).

The cardiovascular and musculoskeletal benefits offered by BFR aerobic training warrant an examination to determine whether it is indeed possible to perform bodyweight jumping with BFR in simulated lunar gravity. If the acute physiological responses closely resemble those observed during BFR aerobic exercise in terrestrial settings (e.g., BFR cycling), including elevated cardiovascular responses (heart rate, oxygen consumption, carbon dioxide production and minute ventilation), metabolic stress (blood lactate and reduced muscle oxygen saturation), impaired post‐exercise force production and greater perceptual demands (exertion and pain), there is a case to be made that similar long‐term training adaptations may alsobe realised for BFR aerobic jumping (Kilgas et al., 2022).

The present study, therefore, aimed to investigate the acute physiological effects of low‐intensity bodyweight jumping in simulated lunar gravity, with and without BFR. It was hypothesised that jumping with BFR, compared to without BFR, will significantly: (1) increase blood lactate concentration, (2) reduce muscle tissue oxygenation of the vastus lateralis (VL) and gastrocnemius medialis (GM), (3) elevate oxygen consumption, carbon dioxide output, breathing frequency and minute ventilation, (4) increase perceived exertion and discomfort, and (5) have no effect on exercise enjoyment or movement instability.

## 2 METHODS

### 2.1 Participants

Fourteen healthy adults participated in the study. Table 1 displays participant characteristics. Sample size was determined from a previous study comparing low‐intensity cycling (40% ${\dot{V}}_{O_{2}\mathrm{peak}}$) with and without BFR that employed a similar exercise protocol and outcome measurements as described in the present study (Kilgas et al., 2022). Effect sizes (Cohen's *d*, mean difference/pooled standard deviation) between BFR (60% LOP) and non‐BFR conditions for each exercise set were computed and converted to Cohen's *f* (√(*d* ^2^/2*k*), where *k* is the number of groups). The average effect size across exercise sets were as follows: tissue oxygen saturation (*f* = 0.59), oxygen uptake (*f* = 0.13), heart rate (*f* = 0.77), respiratory exchange ratio (*f* = 0.39), minute ventilation (*f* = 0.45), rating of perceived exertion (*f* = 0.77), blood lactate (*f* = 0.72) and post‐exercise isometric torque production (*f* = 0.72) (Kilgas et al., 2022). A medium effect size (*f* = 0.25; equivalent to a partial eta squared of 0.06) was selected as a conservative power estimate for the present investigation. Power analysis was performed in G*power (Version 3.1.9.2) in which a sample size of *n* = 14 was required for a two‐way repeated measures ANOVA using the greatest number of pairwise comparisons planned in the present study (2 conditions [BFR vs. non‐BFR] × 11 time ‐points [6 exercise sets + 5 rest periods]) with an estimate effect of *f* = 0.25, type I (α) error of 5%, type II (β) error of 20%, correlation among repeated measures of 0.5 and non‐sphericity correction of 1.0. All participants were informed of the study protocol in advance and provided written consent prior to taking part in the study. The study conformed to the standards set by the 2013 *Declaration of Helsinki* (except pre‐registration) and received ethical approval by Northumbria University (Reference ID: 1993).

**TABLE 1:** Participant characteristics.

Characteristic	Study cohort (*n* = 14)
Male:female (*n*)	11:3
Age (years)	24 ± 4
Height (m)	1.81 ± 0.06
Body mass (kg)^#^	75 ± 12
Systolic blood pressure (mmHg)	129 ± 11
Diastolic blood pressure (mmHg)	76 ± 8
Resting heart rate (beats min^−1^)	65 ± 13
Left thigh limb occlusion pressure (mmHg)	200 ± 18
Right thigh limb occlusion pressure (mmHg)	194 ± 15
${\dot{V}}_{O_{2}\mathrm{peak}}$ (ml kg^−1^ min^−1^)	50 ± 11

*Notes*: Data are means ± standard deviation.

^#^ As measured in the familiarisation visit.

Participants were injury‐free and did not have any known form of cardiovascular, respiratory, metabolic, neural or musculoskeletal disease. Participants were not currently taking any medication or nutritional supplements that could influence the study outcomes, were free from illness in the previous month, did not smoke, did not have any known blood‐borne diseases and did not present any contraindications to exercise or BFR exercise as per a Physical Activity Readiness Questionnaire and BFR Exercise Medical Screening Tool, respectively. Prior to taking part in the study, no participant had ever used BFR as part of a training programme, six had previous experience with BFR in other research projects, and eight had never experienced BFR exercise.

### 2.2 Experimental design

Participants attended a familiarisation session to complete an incremental jumping test in simulated lunar gravity (4‐min stages at increasing jump heights) followed by a graded exercise test (GXT) to volitional exhaustion on a cycle ergometer (upright posture) to obtain maximal oxygen uptake (${\dot{V}}_{O_{2}\mathrm{peak}}$). Linear regression was performed between jump height and steady‐state oxygen uptake (%${\dot{V}}_{O_{2}\mathrm{peak}}$) at each stage to identify the jump height associated with 40% ${\dot{V}}_{O_{2}\mathrm{peak}}$ to prescribe the jumping workload for the BFR and non‐BFR trials. The rationale for prescribing jumping exercise intensity relative to ${\dot{V}}_{O_{2}\mathrm{peak}}$ obtained from a cycling GXT is due to it presently being unknown whether ${\dot{V}}_{O_{2}\mathrm{peak}}$ can be achieved jumping in simulated lunar gravity. In addition, in‐flight aerobic exercise intensities onboard the ISS are prescribed from a pre‐flight cycling graded exercise test on Earth (Loehr et al., 2015). The second and third visits involved the completion of a low‐intensity (40% ${\dot{V}}_{O_{2}\mathrm{peak}}$) jumping session in simulated lunar gravity comprising 6 × 2‐min sets of continuous bilateral countermovement jumping with 1‐min of passive rest between sets. One visit was performed with BFR applied bilaterally on the lower limbs at 60% LOP during the exercise periods and deflated during the rest periods; the BFR visit was counterbalanced among participants to minimise trial‐order effects. Blood lactate was measured at rest and immediately upon completion of each jumping set. Pulmonary gas exchange, heart rate, tissue oxygenation index (VL and GM) and vertical ground reaction forces were measured throughout the jumping exercise period. Rating of perceived exertion, discomfort and movement instability were recorded following completion of each jumping set. Exercise enjoyment was measured post‐exercise via a physical activity enjoyment scale.

Participants were instructed to refrain from alcohol and recreational stimulants (e.g., caffeine) on the day of testing and vigorous exercise <48 h before testing. Participants wore unrestrictive sports clothing (T‐shirt and shorts) and the same pair of trainers for each visit. All sessions were separated by at least 48 h and performed in an environmentally controlled laboratory (temperature: 19.3 ± 0.6°C, humidity: 52 ± 8%, pressure: 1040 ± 9 hPa). The BFR and non‐BFR jumping visits were performed at a similar time of day (±1 h) to minimise the effects of circadian rhythms.

### 2.3 Procedures

#### 2.3.1 Hypogravity analogue

The Variable Gravity Suspension System (VGSS) was employed to simulate lunar gravity. A technical report of the VGSS can be found elsewhere (Swain et al., 2025c). The VGSS enables simulated hypogravity through head‐up tilt bodyweight suspension. In the present study, participants were suspended in a 9.5° head‐up tilt position to allow for ∼17% of Earth's gravity to act along the longitudinal axis of the body (*mg* sin(θ), where *m* is body mass, *g* is Earth's gravitational field strength [9.81 m s^−2^], and θ is head‐up tilt angle relative to the horizontal plane). Participants were suspended in the VGSS by a series of ropes attached to ankle and knee slings, and a custom pelvic and thorax/head support plate, with the angles of each rope set to 9.5° from the vertical plane (i.e., perpendicular to the longitudinal axis of the participant's body). The angle of the suspension ropes and longitudinal axis of the body was verified using a digital inclinometer (RS PRO 175 mm LCD, RS Components, London, UK) to the nearest 0.5°. Each suspension rope passed upward toward a pulley on their respective overhead gantry that passed to the rear of the VGSS and connected to a spring‐balance (Yale YBF Series Spring Balancers, Greenville, NC, USA) to counterbalance the weight of the limb, ensuring the leg did not fall/raise when leaving the foot platform during jumping. A treadmill (Floatride, Reebok, Boston, MA, USA) mounted onto the VGSS served as the foot platform (51 × 150 cm) on which participants stood and jumped. The treadmill orientation was set parallel to the suspension ropes with the belt fixed in place to prevent movement. A 15–30 s period of quiet standing was performed upon suspension for the measurement of ground reaction forces to verify the level of simulated hypogravity.

#### 2.3.2 Incremental jumping test

The incremental jumping protocol has been described in a previous publication (Swain et al., 2025b). The test comprised 4‐min stages of continuous bilateral bodyweight countermovement jumping in simulated lunar gravity, starting at a jump height of 30 cm, increasing by 5 cm per stage, up to a maximum jump height of 70 cm. Jump heights >70 cm were restricted by headroom availability of the VGSS. A 1‐min passive (standing) rest period separated each jumping stage. The test was terminated when the participant could not complete a given stage due to fatigue, was unable to reach the target height, or was unable to jump continuously without stopping (e.g., due to instability). Participants were instructed to jump with relatively stiff legs and to minimise ground contact time to avoid excessive hip/knee flexion motion. Arm swinging was not permitted, and participants were asked to hold onto the back support plate suspension ropes located to each side of the hip. Participants were familiarised with the jumping technique on the day of testing by jumping at heights up to that of the first stage of the incremental test until the participant was comfortable with the jumping technique, which was subsequently approved by a researcher, a process that took ∼5–10 min.

The displacement of the participant in line with the longitudinal axis of their body was recorded via a linear cable extension potentiometer (SP1‐50, Multicomp Pro, Leeds, UK) attached between an aluminium strut profile at the level of the treadmill surface and hip region of the back‐support plate. The signal from the cable extension potentiometer was sampled by an analogto digital modular data acquisition system (NI‐9174, National Instruments, Austin, TX, USA) via an analog input module (NI‐9201, National Instruments), at 2000 Hz, and captured and stored (in .txt format) by a custom program (LabVIEW 2018, National Instruments). The cable extension potentiometer signal was zeroed to the standing position of the participant in simulated lunar gravity across a 10 s period. Jump height was displayed on a graph in real time on a monitor positioned directly above the participant's head in their field of view, with the target jump height denoted by a horizontal line.

#### 2.3.3 Graded exercise test

A standard ramp graded exercise test (GXT) was performed upright (1 *G*_z_) on an electronically braked cycle ergometer (Velotron, SRAM, IL, USA) to determine ${\dot{V}}_{O_{2}\mathrm{peak}}$. The GXT began at 50 W and increased linearly by 0.42 W s^−1^ (25 W min^−1^). Participants were instructed to cycle at 70–90 rpm for as long as possible until volitional exhaustion or inability to maintain a cycling cadence ≥70 rpm. The achievement of ${\dot{V}}_{O_{2}\mathrm{peak}}$ was determined if the linear slope of the ${\dot{V}}_{O_{2}}$–workload relationship over the final 50 W of the GXT was <5 mL min^−1^ W^−1^, which has been demonstrated to reduce the risk of false‐plateau diagnosis to ≤5% (Niemeyer et al., 2020). The absence of a ${\dot{V}}_{O_{2}}$ plateau in GXTs is common, and therefore secondary maximal effort criteria recommended by Wagner and colleagues were employed (Wagner et al., 2020). These included achieving one of the following criteria: (1) RER_max_ ≥ 1.13, (2) age‐predicted maximal heart rate (APMHR) (210 − age) ≥96%, or (3) APMHR (208–0.7 x age) ≥93%. One participant failed to meet any of the above criteria and therefore was invited to re‐perform the GXT on a separate day where they subsequently were able to meet at least one criterion. The highest 15‐breath ${\dot{V}}_{O_{2}}$ average during the GXT was used to define ${\dot{V}}_{O_{2}\mathrm{peak}}$.

#### 2.3.4 Jumping exercise protocol

**FIGURE 1:**
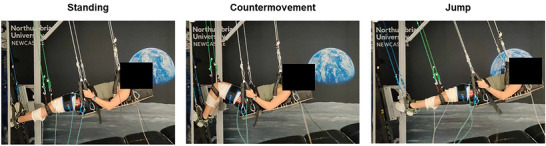
Mock‐up of a user jumping in simulated lunar gravity within the variable gravity suspension system.

The BFR and non‐BFR jumping protocol involved a 3‐min warm‐up at the jump height associated with 40% ${\dot{V}}_{O_{2}\mathrm{peak}}$ (no BFR was applied during the warm‐up period in either session) in accordance with ‘low‐intensity’ BFR aerobic training protocols performed at metabolic rates <50% ${\dot{V}}_{O_{2}\mathrm{peak}}$ (Patterson et al., 2019). Participants then performed 6 × 2‐min sets of continuous bilateral countermovement jumping at the same height with 1‐min passive inter‐set rest periods. The protocol was selected to allow for comparison with the recent study of Kilgas et al. (2022) that investigated the acute physiological responses to 6 × 2‐min of BFR cycling at 40% ${\dot{V}}_{O_{2}\mathrm{peak}}$ and reported similar outcomes to those measured in the present study. Jumping frequency was standardised to the participants’ preferred rate of jumping at the target jump height determined during the 3‐min warm‐up period of the first jumping exercise session using a metronome. The metronome was set at this pace for both BFR and non‐BFR jumping conditions. Participants received the same jump height visual feedback as for the incremental jumping test. The participant was instructed to jump continuously at the target jump height and metronome frequency for each exercise set. Figure 1 illustrates a user jumping in simulated lunar gravity with BFR. Supporting information Video S1 demonstrates a user jumping in simulated lunar gravity within the VGSS.

#### 2.3.5 Blood flow restriction

Blood flow restriction was applied bilaterally using the Delfi Personalized Tourniquet System for BFR (Delfi Medical Innovations Inc, Vancouver, BC, Canada). Full details of the BFR device and procedures can be found in Supporting information, Table S1 in accordance with the recent editorial concerning the reporting of BFR apparatus and features (Hughes et al., 2025). The BFR system comprised two pneumatic tourniquet cuffs connected to a pneumatic device which has been demonstrated to autoregulate cuff pressure within approximately ±10% of the target pressure during lower limb exercise (Hughes et al., 2024; Swain et al., 2025a). The tourniquet cuffs were applied proximally on both lower limbs. Limb occlusion pressure was automatically measured on the proximal portion of the left and right thigh for each participant using the BFR system that has been validated against the previous gold‐standard Doppler‐ultrasound technique (Masri et al., 2016). The LOP measurement was performed with the participant quietly standing in the 9.5° head‐up tilt suspended position prior to commencing the jumping exercise to control for the effect of body orientation, with respect to gravity, on LOP (Hughes et al., 2018; Swain et al., 2024). The pneumatic tourniquet cuffs were inflated to 60% LOP during the 2‐min jumping periods and fully deflated during each 1‐min rest period (Patterson et al., 2019).

#### 2.3.6 Knee extension force

Peak force during maximal voluntary isometric contraction (MVIC) of the knee extensors (left leg) was assessed pre‐ and post‐exercise as a marker of acute exercise‐induced fatigue. Participants sat upright on a high‐seated flat‐bottomed chair with the left knee extended at 110 ± 5° (verified via a goniometer). A padded strap was secured around the lowest portion of the leg, above the ankle joint, which connected to a strain gauge via carabiners in the horizontal plane (MIE Myometer, MIE Medical Research Ltd, Leeds, UK). The strain gauge was zeroed with the leg at rest. Six warm‐up repetitions were performed, two of each at 25%, 50% and 75% of the participant's perceived maximal effort (30 s between repetitions). A 2‐min passive rest followed the final warm‐up repetition. Participants then performed 3 × 5 s MVICs with verbal encouragement from the researcher, separated each by 2 min of passive rest. Post‐exercise MVICs were initiated at +5 min following the completion of the final jumping set due to the time required to de‐suspend the participant from the VGSS and set‐up on the knee extension chair. The peak force values achieved for each of the three pre‐ and post‐exercise MVICs were averaged for analysis.

### 2.4 Measurements

#### 2.4.1 Body mass and height

Body mass and height were measured using a digital scale to the nearest 0.1 kg (Seca 703 Column Scale, Hamburg, Germany) and stadiometer to the nearest 0.01 m (Seca 213 Portable Height Measurer, Hamburg, Germany), respectively.

#### 2.4.2 Force platform and cable extension potentiometer

Two force platforms (OR6‐7, AMTI, Watertown, MA, USA) embedded in parallel directly underneath the VGSS treadmill measured ground reaction forces. Signals from the force platform passed to a strain gauge amplifier (MSA‐6, AMTI) with the gain of the vertical force component set to 4000. The amplified signal was sampled by the same data acquisition hardware and custom program as the cable extension potentiometer. The force platforms were hardware zeroed prior to each test at the 9.5° tilt angle on each testing day. Filters were applied to the raw force data (50 Hz low‐pass fourth‐order Butterworth) and cable extension potentiometer data (20 Hz low‐pass fourth‐order Butterworth). The force platform and suspended participant should, in an ideal scenario, be perpendicular; however, due to slight variability in suspension position and foot placement during jump landings relative to the force plate, a component of vertical ground reaction force (vGRF) (*F*_z_) along the longitudinal axis of the body can be measured in the anterior‐posterior axis (*F*_y_). To account for this, vGRF was computed as the resultant of the *F*_z_ and *F*_y_ forces in accordance with the Pythagorean theorem. For each individual jump, the height and depth of the jump (i.e., maximum and minimum cable extension potentiometer values, respectively), time period between jumps, and peak vGRF (i.e., highest vGRF value) were identified post‐filtering. The average jump height, frequency, depth and peak vGRF were computed for each set of jumping exercise. Force data are expressed as a percentage of 1 *G*_z_ bodyweight. Pre‐processing and analysis of the force and cable potentiometer data were performed using custom MATLAB code (MATLAB 2023b, MathWorks, Natick, MA, USA)

#### 2.4.3 Near‐infrared spectroscopy

Local changes in muscle tissue oxygenation at the VL and GM were determined using multi‐distance continuous‐wave near‐infrared spectroscopy (NIRS) at 5 Hz (NIRO‐200NX, Troy, MI, USA). The skin overlaying the muscle regions of interest was shaved (if necessary) and cleaned with a 70% isopropyl alcohol wipe to improve sensor‐skin adherence. The sensor for the VL was placed 67% along the line between the anterior superior iliac spine and lateral patella. The sensor for the GM was placed on the most prominent bulge of the muscle. The infrared light emitting diode was always positioned proximally to the photodetector and aligned to the longitudinal axis of the muscle. Each sensor was held in place by double‐sided tape, with additional micropore tape overlaid and covered by a black rubber casing and an opaque elasticised bandage to reduce sensor movement and interference from ambient lighting. The sensors were placed on the left leg for all participants due to it being the most accessible side of the body when suspended, minimising practical issues with cable management. Before the warm‐up, participants stood quietly in simulated lunar gravity for 5 min before initiating the NIRS recording, after which a further 5 min of standing was used to establish a resting baseline average. A 5 s rolling‐average was applied to the raw data and the change in tissue oxygenation index (ΔTOI) during each exercise and rest period was determined as the average of the final 15 s minus the 5 min baseline average.

#### 2.4.4 Pulmonary gas exchange

Expired gas was measured on a breath‐by‐breath basis using a metabolic cart (MetaLyzer 3B, Cortex Medical, Leipzig, Germany) and associated software (MetaSoft Studio, Cortex Medical). The MetaLyzer was calibrated using a known gas volume (3 L syringe), concentration of O_2_ (15.08%) and CO_2_ (5.03%), and ambient air on each testing day. Heart rate (HR) was measured via a Polar heart rate sensor (Polar H9, Kempele, Finland) secured around the chest of the participant at the level of the xiphoidal process. Heart rate data were transmitted to the MetaSoft Studio software wirelessly via receiver using an existing MetaLyzer plug‐in. The following outcomes were analysed: breathing frequency (BF), minute ventilation (${\dot{V}}_{E}$), volume of oxygen consumption (${\dot{V}}_{O_{2}}$), volume of carbon dioxide output (${\dot{V}}_{CO_{2}}$) and HR. A 15‐breath rolling average aligned to the central (eighth) breath was applied to the raw data in accordance with indirect calorimetry data processing guidelines (Robergs et al., 2010). The final 15‐breath average for each set of exercise and recovery period was taken for analysis.

#### 2.4.5 Blood lactate

Capillary blood lactate (BLA) samples were collected at rest and immediately after the completion of each exercise set. The finger or earlobe (depending on participant preference) was cleaned with an 70% isopropyl alcohol wipe and punctured with a disposable lancet device (Accu‐Chek, Roche Diagnostics, West Sussex, UK). The first blood droplet was removed, and the second was collected in a 20 µL capillary tube and placed in a reaction cup pre‐filled with 1000 µL of haemolysing solution for mixing via several slow inversions. Samples were analysed using the Biosen C‐Line Glucose and Lactate Analyzer (EKF, Stroke on Trent, UK), calibrated with a multi‐standard lactate and glucose solution as per the manufacturer's instructions.

#### 2.4.6 Perceived exertion, discomfort and body instability

The participant was asked to score three scales relating to perceived exertion, body control and discomfort immediately following each set of jumping. The Borg rating of perceived exertion (RPE) chart was used to determine subjective levels of task exertion ranging from a score of 6 (‘no exertion’) to 20 (‘maximal exertion’) (Borg, 1998). Discomfort was assessed using the Borg‐CR10 scale ranging from 0 ‘nothing at all’ to 11 ‘absolute maximum’. The Modified Cooper–Harper Body Control Scale was used to gauge body control during jumping within the BWS ranging from a score of 1 (unrestricted) to 10 (body control lost). To avoid increases in body control being misinterpreted as improved body control, the term ‘body instability’ has been used.

#### 2.4.7 Physical activity enjoyment

The 16‐item Physical Activity Enjoyment Scale (PACES) was completed by participants following the post‐exercise knee extension fatigue assessment (∼10 min after completing the jumping exercise) (Motl et al., 2001). The PACES tool has been established as reliable and valid in adults (Jekauc et al., 2020) and has previously been employed to compare exercise enjoyment between different exercise protocols (e.g., moderate‐ versus high‐intensity exercise) (Thum et al., 2017). The PACES questionnaire required the participant to score 16 questions worded positively and negatively regarding how enjoyable the completed physical activity was using a 5‐point Likert scale (1 = strongly disagree; 5 = strongly agree). Negatively worded items were re‐coded to fit with the positively worded scale and the average of item scores was calculated for each participant with higher scores being indicative of greater perceived enjoyment (Jekauc et al., 2020).

### 2.5 Statistical analyses

SPSS Statistics, Version 29 (IBM Corp., Armonk, NY, USA) was used for statistical analyses. Standing vGRF within the VGSS was compared between the familiarisation, non‐BFR and BFR visits via a one‐way repeated measures analysis‐of‐variance (RM‐ANOVA). A paired sample Student's *t*‐test was used to compare jump height and frequency and cardiovascular exercise intensity (% ${\dot{V}}_{O_{2}\mathrm{peak}}$) during the 3 min jumping warm‐up between conditions. Two‐way RM‐ANOVAs examined the effect of jumping condition (BFR vs. non‐BFR) at each measurement time point for jump height, depth, frequency and peak vGRF, HR, ${\dot{V}}_{O_{2}}$, ${\dot{V}}_{CO_{2}}$, BF, ${\dot{V}}_{E}$, VL and GM ΔTOI, and blood lactate concentration. Jump height, depth, frequency, peak vGRF and perceptual outcomes included one time point per exercise set (×6); blood lactate comparisons also included a baseline (resting) timepoint. The other listed outcomes included a measure for each exercise set (×6) and rest period (×5). Knee extension peak force pre‐ and post‐exercise between BFR and non‐BFR conditions was also assessed using a two‐way RM‐ANOVA. Data were assessed for outliers, normality (Shapiro–Wilk test, skewness and kurtosis, and Q‐Q plots), and sphericity (Mauchly's test of sphericity) to determine appropriateness of data transformation or non‐parametric test equivalents. Supporting information Table S2 includes a statistical analysis summary report including data exclusions and transformations. The Greenhouse–Geisser correction was used if sphericity was violated in the ANOVA models. Simple main effects were examined using *post hoc* comparisons with Bonferroni adjustments. Perceptual responses were measured using ordinal scales, and therefore were compared between conditions using the non‐parametric Wilcoxon signed‐rank test (physical activity enjoyment) and Friedman's test (PRE, discomfort and movement instability). Significance in the Friedman's test was followed up by a *post hoc* Wilcoxon signed‐rank test to compare responses between the non‐BFR and BFR conditions for each jumping exercise set; *P*‐values were adjusted in accordance with the Bonferroni method.

Test–retest reliability between BFR and non‐BFR visits during the 3‐min warm‐up period was assessed using intra‐class correlation coefficients (ICCs; two‐way mixed; absolute agreement; average measures) for jump height, depth, jump frequency, peak vGRFs, cardiorespiratory measures, and VL and GM ΔTOI. Pre‐exercise knee extension MVIC peak force was compared between sessions. The following reliability descriptors were used: <0.5 (poor), 0.5–0.74 (moderate), 0.75–0.90 (good) and >0.90 (excellent) (Koo and Li, 2016). Partial eta squared (η_p_^2^) is reported for repeated measures analyses as calculated in SPSS (Cohen, 1988). Effect sizes (Cohen's *d*) are reported for pairwise comparisons. Statistical significance was set to *P* < 0.05. Data are reported as means ± standard deviation unless otherwise stated.

## 3 RESULTS

Participants completed all sets of jumping during the BFR and non‐BFR exercise sessions; no adverse events were reported. The level of partial weight‐bearing (% bodyweight) was not different between conditions and verified participants were within simulated lunar gravity: 17 ± 3% (familiarisation), 19 ± 3% (non‐BFR control) and 18 ± 2% (BFR) (*P* = 0.269). During the 3‐min warm‐up period, in which participants jumped at a pre‐defined jump height corresponding to 40% ${\dot{V}}_{O_{2}\mathrm{peak}}$ without BFR, participants successfully jumped at similar heights and frequencies between conditions: 38 ± 6 cm (non‐BFR session) and 38 ± 6 cm (BFR session) (*P* = 0.664) and 35 ± 4 jumps/min (non‐BFR session) and 36 ± 3 jumps/min (BFR session) (*P* = 0.347). In addition, participants jumped close to the target exercise intensity of 40% ${\dot{V}}_{O_{2}\mathrm{peak}}$ in both sessions: 37 ± 4% ${\dot{V}}_{O_{2}\mathrm{peak}}$ (non‐BFR session) and 39 ± 5% ${\dot{V}}_{O_{2}\mathrm{peak}}$ (BFR session) (*P* = 0.0789). Each participant successfully completed the BFR jumping exercise at 60% LOP in all but one case in which pressure was reduced to 50% LOP for sets 3–6 to ensure the participant could maintain the target jump height due to fatigue/discomfort. Table 2 displays the between‐session reliability of outcome measurements collected during the 3‐min warm‐up jumping period.

**TABLE 2:** Reliability of measures during jumping in simulated lunar gravity.

Measurement	Warm‐up		Mean difference [95% CI]	ICC [95%CI]	*P*
	Non‐BFR visit	BFR visit			
Jump characteristic					
Jump height (cm)	38 ± 6	38 ± 6	0 [−1, 1]	0.99 [0.98, 0.99]	<0.001
Jump frequency (jumps min^−1^)	35 ± 4	36 ± 3	−1 [−2, 1]	0.85 [0.54, 0.95]	<0.001
Jump depth (cm)	−28 ± 8	−29 ± 9	1 [−2, 3]	0.93 [0.79, 0.98]	<0.001
Peak vGRF (% bodyweight)	66 ± 19	74 ± 17	−8 [−19, 2]	0.61 [−0.08, 0.87]	0.0381
∆TOI (% baseline)					
Vastus lateralis (%)	−8 ± 4	−10 ± 4	2 [1, 3]	0.87 [0.29, 0.96]	<0.001
Gastrocnemius medialis (%)^#^	3 ± 5	2 ± 4	1 [−2, 4]	0.72 [−0.03, 0.92]	0.0311
Cardiorespiratory					
Heart rate (b min^−1^)^*^	95 ± 8	99 ± 12	−4 [−7, −1]	0.91 [0.53,0.98]	<0.001
${\dot{V}}_{O_{2}}$ (ml kg^−1^ min^−1^)	18 ± 3	19 ± 4	−1 [−2, 1]	0.90 [0.67, 0.97]	<0.001
${\dot{V}}_{CO_{2}}$ (ml kg^−1^ min^−1^)	16 ± 3	17 ± 4	−1 [−2, 0]	0.91 [0.69, 0.97]	<0.001
BF (n min^−1^)	29 ± 5	29 ± 6	0 [−3, 3]	0.78 [0.29, 0.93]	0.00601
${\dot{V}}_{E}$ (L min^−1^)	34 ± 7	36 ± 8	−3 [−5, 0]	0.90 [0.60, 0.97]	<0.001
Isometric force (pre‐exercise)
Knee extension peak force (kg)	62 ± 15	63 ± 16	−1 [−5, 4]	0.94 [0.83, 0.98]	<0.001

*Note*: Data are means ± standard deviation. The 3‐min warm‐up period during both visits included jumping at the same height and frequency (corresponding to 40% ${\dot{V}}_{O_{2}\mathrm{peak}}$) without blood flow restriction. Values have been rounded. Sample sizes are *n* = 14 except for gastrocnemius medialis ∆TOI (^#^ *n* = 11 due to poor signal quality) and heart rate (^*^ *n* = 12 due to signal loss). The *P*‐values correspond to the ICC model. Values have been rounded.

Abbreviations: BF, breathing frequency; BFR, blood flow restriction; CI, confidence interval; ICC, intraclass correlation coefficient; TO, tissue oxygenation index; ${\dot{V}}_{CO_{2}}$, volume of carbon dioxide output; ${\dot{V}}_{E}$, minute ventilation; vGRF, vertical ground reaction force; ${\dot{V}}_{O_{2}}$, volume of oxygen consumption.

### 3.1 Jump height, frequency, depth and peak vertical ground reaction force

Table 3 displays the height, frequency, depth and peak vGRFs of jumping in simulated lunar gravity with and without BFR. No significant effect for condition, time, or condition–time interaction was observed for jump height, frequency or depth. Peak vGRFs revealed a significant effect for condition (∼10% bodyweight higher during BFR jumping) and time (no interaction effect), increasing by 8–9% bodyweight across jumping sets one through six in both conditions.

**TABLE 3:** Jump height, frequency, depth, and peak vertical ground reaction forces during BFR and non‐BFR jumping in simulated lunar gravity.

Measurement	Set 1	Set 2	Set 3	Set 4	Set 5	Set 6	Two‐way ANOVA		
							Test of effect	*P*	η_p_^2^
Jump height (cm)									
Non‐BFR	39 ± 6	39 ± 7	39 ± 7	39 ± 7	39 ± 7	39 ± 7	Condition	0.539	0.030
BFR	39 ± 7	39 ± 7	39 ± 7	38 ± 7	38 ± 7	39 ± 7	Time	0.291	0.091
							Interaction	0.365	0.078
Jump frequency (jumps per minute)									
Non‐BFR	36 ± 3	36 ± 4	36 ± 4	36 ± 4	36 ± 4	36 ± 4	Condition	0.160	0.146
BFR	36 ± 4	36 ± 4	36 ± 4	37 ± 4	36 ± 4	37 ± 4	Time	0.747	0.018
							Interaction	0.185	0.118
Jump depth (cm)									
Non‐BFR	−29 ± 9	−29 ± 10	−29 ± 9	−30 ± 9	−30 ± 10	−31 ± 10	Condition	0.667	0.015
BFR	−29 ± 10	−30 ± 11	−29 ± 12	−29 ± 12	−29 ± 12	−29 ± 13	Time	0.538	0.046
							Interaction	0.194	0.115
Peak vertical ground reaction force (% bodyweight)									
Non‐BFR	72 ± 18	76 ± 18	77 ± 19	76 ± 21	77 ± 20	78 ± 23	Condition	0.00258	0.515
BFR	81 ± 20	86 ± 21	89 ± 24	88 ± 23	87 ± 23	90 ± 24	Time	0.0102	0.275
*P*	0.0266	0.0108	0.00151	0.00609	0.0129	0.00485	Interaction	0.687	0.032
ES	0.47	0.51	0.55	0.54	0.46	0.51			

*Note*: Data are mean ± standard deviation. Sample sizes are *n* = 14. Abbreviations: BFR, blood flow restriction, ES, effect size (Cohen's *d*).

### 3.2 Vastus lateralis and gastrocnemius medialis tissue oxygenation index

**FIGURE 2:**
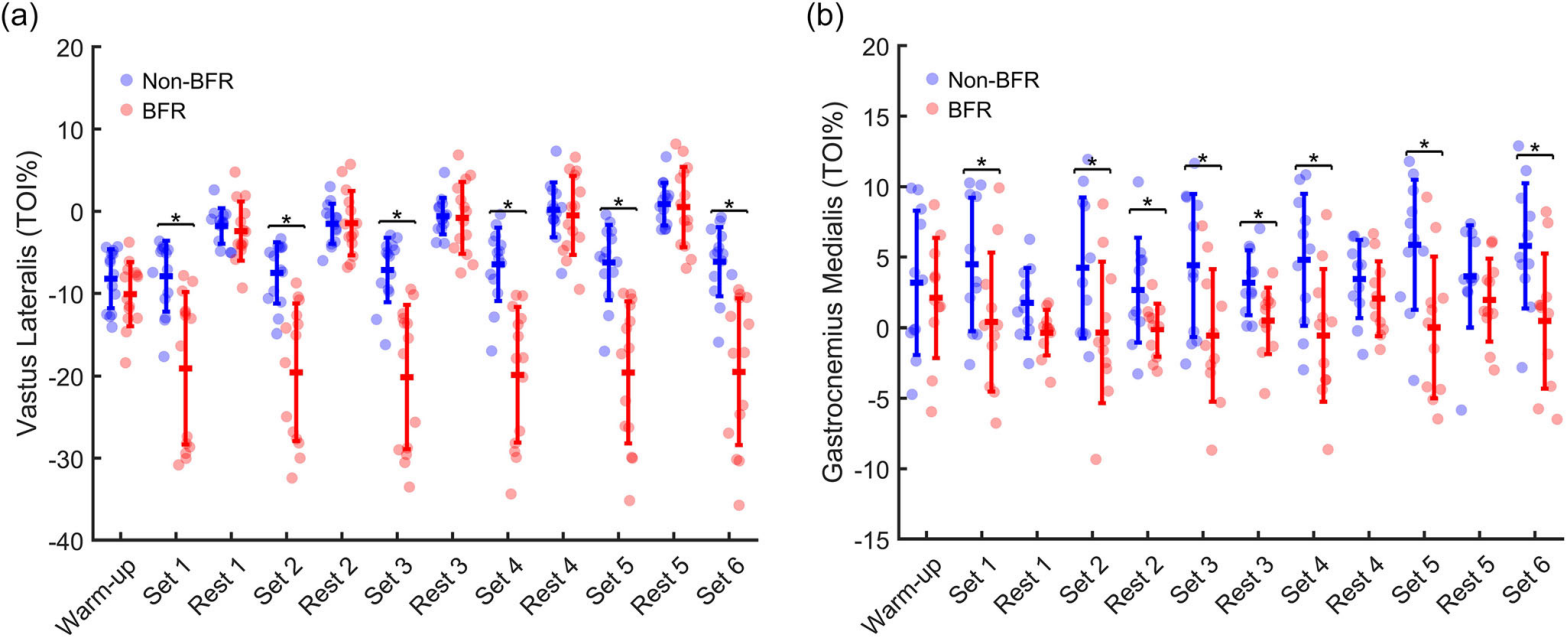
Tissue oxygenation index (∆TOI; % baseline) at the vastus lateralis (a; *n* = 14) and gastrocnemius medialis (b; *n* = 11) between BFR and non‐BFR jumping conditions in simulated lunar gravity. Data are means ± standard deviation. *Between‐condition significance (*P* < 0.05). See Table 4 for all between‐condition *P*‐values.

Table 4 and Figure 2 display ∆TOI (% baseline) responses for the VL and GM during jumping in simulated lunar gravity with and without BFR. Jumping with BFR caused VL TOI to significantly decline to ∼20% below resting baseline levels during each jumping set compared to without BFR (∼5–8%). During the 1‐min recovery periods, VL TOI returned to approximately resting values and did not significantly differ between BFR and non‐BFR jumping conditions. Data from three participants were excluded for GM TOI due to poor signal quality. The GM displayed a mean increase in TOI above baseline values by ∼5% during non‐BFR jumping that was significantly attenuated with the application of BFR in which TOI remained close to resting values; however, it is worth noting that the individual GM TOI responses were heterogeneous.

**TABLE 4:** Tissue oxygenation index of the vastus lateralis and gastrocnemius medialis during non‐BFR and BFR jumping in simulated lunar gravity.

	Tissue Oxygenation Index (% change from baseline)													
Tissue region	Jump set 1		Jump set 2		Jump set 3		Jump set 4		Jump set 5		Jump set 6	Two‐way ANOVA		
	Exercise	Rest	Exercise	Rest	Exercise	Rest	Exercise	Rest	Exercise	Rest	Exercise	Test of Effect	*P*	η_p_^2^
Vastus lateralis (*n* = 14)														
Non‐BFR	−8 ± 4	−2 ± 2	−8 ± 4	−2 ± 2	−7 ± 4	−1 ± 2	−6 ± 4	−0 ± 3	−6 ± 5	−1 ± 3	−6 ± 4	Condition	<0.001	0.846
BFR	−19 ± 9	−2 ± 4	−20 ± 8	−1 ± 4	−20 ± 9	−1 ± 4	−20 ± 8	−1 ± 5	−20 ± 9	1 ± 5	−20 ± 9	Time	<0.001	0.808
*P*	<0.001	0.593	<0.001	0.949	<0.001	0.882	<0.001	0.696	<0.001	0.815	<0.001	Interaction	<0.001	0.585
ES	1.58	0.00	1.90	0.32	1.87	0.00	2.21	0.24	1.92	0.00	2.01			
Gastrocnemius medialis (*n* = 11)														
Non‐BFR	4 ± 5	2 ± 2	4 ± 5	3 ± 4	4 ± 5	3 ± 2	5 ± 5	3 ± 3	6 ± 5	4 ± 4	6 ± 4	Condition	0.0173	0.447
BFR	0 ± 5	0 ± 2	0 ± 5	0 ± 2	−1 ± 5	0 ± 2	−1 ± 5	2 ± 3	0 ± 5	2 ± 3	0 ± 5	Time	0.177	0.163
*P*	0.0343	0.0535	0.0258	0.0214	0.0131	0.0195	0.0119	0.233	0.0130	0.224	0.0136	Interaction	0.0124	0.317
ES	0.80	1.00	0.80	0.95	1.00	1.50	1.20	0.33	1.20	0.57	1.32			

*Note*: Data are means ± standard deviation. Abbreviations: BFR, blood flow restriction; ES, effect size (Cohen's *d*).

### 3.3 Cardiovascular

Table 5 displays the cardiovascular responses to jumping in simulated lunar gravity with and without BFR. A significant main effect for condition, time and condition–time interaction was observed for ${\dot{V}}_{O_{2}}$ and HR. Minute ventilation and ${\dot{V}}_{CO_{2}}$ revealed a significant effect for time and condition, and no time–condition interaction. Breathing frequency revealed a significant effect for time only. Simple pairwise comparisons revealed ${\dot{V}}_{O_{2}}$ was either unaffected or significantly increased with BFR during some exercise and rest periods by modest degrees (2–3 mL kg^−1^ min^−1^). Carbon dioxide output was significantly elevated at each measurement time point from the first rest period onward by 2–4 mL kg^−1^ min^−1^. Minute ventilation was significantly elevated with BFR from the first rest period onward by 7–16 L min^−1^. Heart rate significantly increased with BFR, relative to without BFR, during the jumping and resting periods by 10–23 beats min^−1^.

**TABLE 5:** Cardiovascular responses to Non‐BFR and BFR jumping in simulated lunar gravity.

Measurement	Jump set 1		Jump set 2		Jump set 3		Jump set 4		Jump set 5		Jump set 6	Two‐way ANOVA		
	Exercise	Rest	Exercise	Rest	Exercise	Rest	Exercise	Rest	Exercise	Rest	Exercise	Test of Effect	*P*	η_p_^2^
${\dot{V}}_{O_{2}}$ (ml kg^−1^ min^−1^)														
Non‐BFR	18 ± 2	12 ± 1	18 ± 2	12 ± 1	18 ± 3	11 ± 1	18 ± 3	12 ± 1	18 ± 3	11 ± 2	18 ± 3	Condition	0.0142	0.406
BFR	17 ± 3	12 ± 2	18 ± 3	13 ± 2	18 ± 3	14 ± 3	19 ± 2	13 ± 3	20 ± 3	13 ± 2	19 ± 3	Time	<0.001	0.884
*P*	0.0958	0.304	0.264	0.144	0.598	0.00361	0.0123	0.0628	0.0545	0.00149	0.167	Interaction	<0.001	0.254
ES	0.39	0.00	0.00	0.63	0.00	1.34	0.39	0.44	0.67	1.00	0.33			
${\dot{V}}_{CO_{2}}$ (ml kg^−1^ min^−1^)														
Non‐BFR	14 ± 2	11 ± 2	15 ± 2	11 ± 2	15 ± 3	11 ± 1	15 ± 3	11 ± 2	15 ± 2	11 ± 2	15 ± 3	Condition	<0.001	0.684
BFR	14 ± 3	13 ± 2	17 ± 3	14 ± 3	17 ± 3	15 ± 3	18 ± 3	14 ± 2	18 ± 3	14 ± 3	18 ± 4	Time	<0.001	0.722
*P*	0.776	0.00269	0.0105	0.00104	0.0239	<0.001	0.0126	<0.001	0.0151	0.00205	0.109	Interaction	0.0589	0.183
ES	0.00	1.00	0.78	1.18	0.67	1.79	1.00	1.50	1.18	1.18	0.85			
${\dot{V}}_{E}$ (L min^−1^)														
Non‐BFR	34 ± 7	27 ± 6	37 ± 8	28 ± 5	37 ± 8	27 ± 4	37 ± 8	28 ± 5	38 ± 8	26 ± 7	38 ± 8	Condition	<0.001	0.702
BFR	36 ± 7	35 ± 8	46 ± 15	36 ± 10	44 ± 13	40 ± 10	50 ± 19	39 ± 10	53 ± 19	38 ± 11	54 ± 23	Time	<0.001	0.566
*P*	0.285	0.00468	0.00459	0.00114	0.0132	<0.001	0.00729	<0.001	0.00175	<0.001	0.00999	Interaction	0.0740	0.167
ES	0.29	1.13	0.75	1.01	0.65	1.71	0.89	1.39	1.03	1.30	0.93			
BF (*n* min^−1^)														
Non‐BFR	28 ± 4	26 ± 4	30 ± 4	27 ± 4	31 ± 5	27 ± 4	31 ± 5	27 ± 3	32 ± 4	26 ± 5	32 ± 5	Condition	0.180	0.134
BFR	29 ± 7	28 ± 5	30 ± 7	29 ± 6	29 ± 8	30 ± 6	32 ± 7	30 ± 6	33 ± 7	29 ± 6	33 ± 7	Time	<0.001	0.444
												Interaction	0.234	0.099
HR (b min^−1^)														
Non‐BFR	96 ± 9	71 ± 12	96 ± 9	74 ± 12	97 ± 9	74 ± 13	98 ± 9	75 ± 12	100 ± 12	77 ± 13	101 ± 12	Condition	<0.001	0.762
BFR	106 ± 15	83 ± 14	113 ± 18	89 ± 17	118 ± 17	92 ± 17	121 ± 19	96 ± 20	122 ± 19	95 ± 18	124 ± 21	Time	<0.001	0.914
*P*	0.0498	0.00120	0.00334	0.00206	<0.001	<0.001	<0.001	<0.001	<0.001	<0.001	<0.001	Interaction	0.00819	0.356
ES	0.81	0.92	1.19	1.02	1.54	1.19	1.55	1.27	1.38	1.15	1.34			

*Note*: Data are means ± standard deviation. Sample sizes are *n* = 14 apart from ${\dot{V}}_{O_{2}}$ and ${\dot{V}}_{CO_{2}}$ (*n* = 13) due to outlier removal, and heart rate (*n* = 11) due to signal loss. Abbreviations: BF, breathing frequency; BFR, blood flow restriction; ES, effect size (Cohen's *d*); HR, heart rate; ${\dot{V}}_{CO_{2}}$, volume of carbon dioxide output; ${\dot{V}}_{E}$, minute ventilation; ${\dot{V}}_{O_{2}}$, volume of oxygen uptake.

### 3.4 Blood Lactate

**FIGURE 3:**
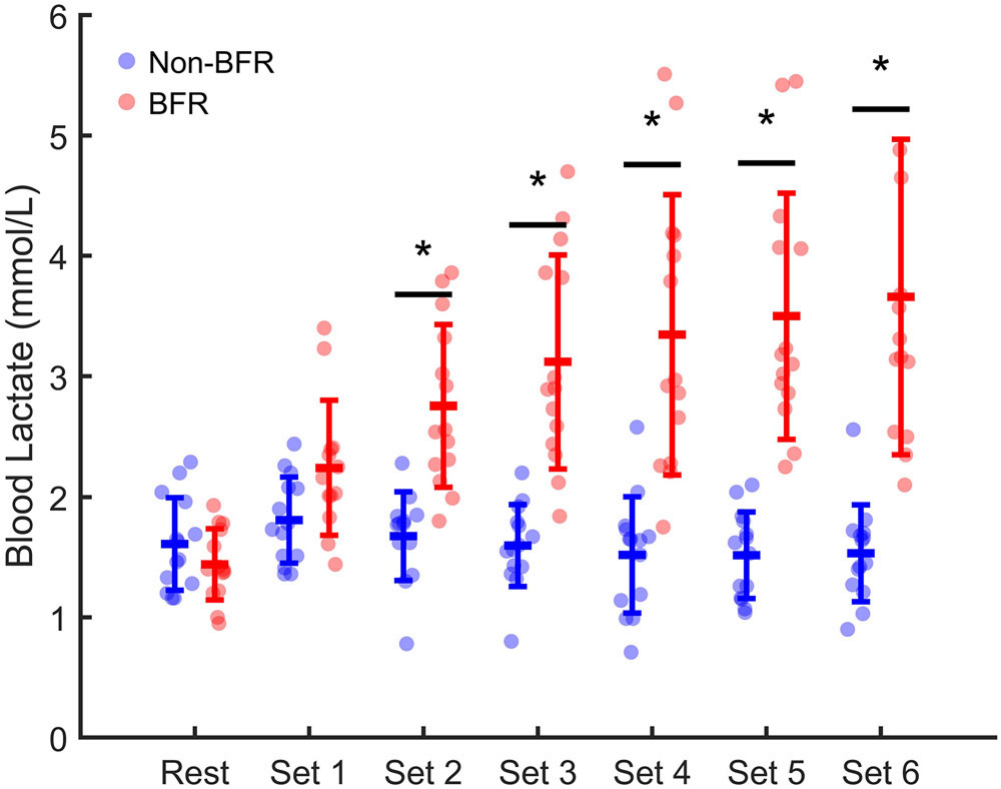
Blood lactate concentrations between BFR and non‐BFR jumping conditions in simulated lunar gravity (*n* = 13). Data are means ± standard deviation. *Between‐condition significance at the corresponding measurement timepoint (*P* < 0.001). See Table 6 for all between‐condition *P*‐values.

Table 6 and Figure 3 display BLA concentrations following each set of jumping in simulated lunar gravity with and without BFR. One participant was excluded from the analysis due to a missing blood sample. Significant main effects for condition, time and condition–time interaction were observed for BLA. Jumping with BFR, compared to without BFR, significantly elevated BLA from the second set of jumping onward. Following completion of the final set of jumping, BLA concentrations were 1.5 ± 0.4 and 3.7 ± 1.3 mmol L^−1^ for non‐BFR and BFR jumping conditions (*P* < 0.001), respectively.

**TABLE 6:** Blood lactate concentrations following each exercise set during non‐BFR and BFR jumping in simulated lunar gravity.

	Blood lactate concentration (mmol/L)							Two‐way ANOVA		
	Resting	Set 1	Set 2	Set 3	Set 4	Set 5	Set 6	Test of effect	*P*	η_p_^2^
Non‐BFR	1.6 ± 0.4	1.8 ± 0.4	1.7 ± 0.4	1.6 ± 0.3	1.5 ± 0.5	1.5 ± 0.4	1.5 ± 0.4	Condition	<0.001	0.854
BFR	1.4 ± 0.3	2.2 ± 0.6	2.8 ± 0.7	3.1 ± 0.9	3.3 ± 1.2	3.5 ± 1.0	3.7 ± 1.3	Time	<0.001	0.555
*P*	0.177	0.0488	<0.001	<0.001	<0.001	<0.001	<0.001	Interaction	<0.001	0.727
ES	0.57	0.78	1.93	2.24	1.96	2.63	2.29			

*Notes*: Data are means ± standard deviation. Sample size is *n* = 13 due to a missing blood sample.

Abbreviations: BFR, blood flow restriction; ES, effect size (Cohen's *d*).

### 3.5 Knee extension peak force

Post‐exercise peak knee extension force declined by 7 ± 9% (BFR) and 4 ± 9% (non‐BFR) (between‐condition Cohen's *d* = 0.33), relative to baseline scores. A significant main effect for time (*P* = 0.00389; η_p_^2^ = 0.001) was observed; however, no significant effects were found for condition (*P* = 0.943; η_p_^2^ = 0.502) or condition–time interaction (*P* = 0.484; η_p_^2^ = 0.037).

### 3.6 Perceptual responses

Table 7 displays perceptual responses to jumping with and without BFR in simulated lunar gravity. Rating of perceived exertion, discomfort and movement quality all revealed a significant main effect from Friedman's test (all *P* < 0.001). *Post hoc* tests revealed that jumping with BFR significantly increased RPE, discomfort and perceived movement instability during each of the six jumping sets compared to without BFR. Physical activity enjoyment (PACES) was not different between non‐BFR (3.9 ± 0.5) and BFR (3.9 ± 0.4) conditions (*P* = 0.944).

**TABLE 7:** Perceptual responses following each exercise set during non‐BFR and BFR jumping in simulated lunar gravity (*n* = 14).

Condition	Set 1	Set 2	Set 3	Set 4	Set 5	Set 6
Rating of perceived exertion (scale 6–20)						
Non‐BFR	7 ± 1	9 ± 2	9 ± 2	10 ± 2	10 ± 2	10 ± 2
BFR	11 ± 2	13 ± 3	15 ± 3	15 ± 2	16 ± 2	16 ± 2
*P*	0.00569	0.00562	0.00579	0.00564	0.00564	0.00567
ES	2.53	1.57	2.35	2.50	3.00	3.00
Discomfort (scale 0–11)						
Non‐BFR	1 ± 1	1 ± 2	1 ± 2	1 ± 2	1 ± 2	1 ± 1
BFR	3 ± 2	4 ± 3	5 ± 3	5 ± 3	6 ± 3	6 ± 3
*P*	0.00864	0.00872	0.0128	0.00870	0.0131	0.00867
ES	1.26	1.50	1.57	1.57	1.96	2.24
Movement instability (scale 0–10)						
Non‐BFR	2 ± 1	2 ± 1	2 ± 1	2 ± 1	2 ± 1	2 ± 1
BFR	3 ± 1	4 ± 1	4 ± 1	4 ± 1	5 ± 1	5 ± 1
*P*	0.0189	0.00731	0.00703	0.00801	0.00801	0.00801
ES	1.00	2.00	2.00	2.00	3.00	3.00

*Note*: Data are means ± standard deviation. *P*‐values from independent Wilcoxon signed‐rank tests have been adjusted using the Bonferroni method. Abbreviations: BFR, blood flow restriction; ES, effect size (Cohen's *d*).

## 4 DISCUSSION

### 4.1 Summary of key findings

This study offers new insights into the acute physiological responses elicited during low‐intensity bodyweight jumping within simulated lunar gravity (∼20% bodyweight), with and without BFR. The key findings were that performing 6 × 2‐min intervals (1‐min rest periods) of bodyweight jumping at 40% ${\dot{V}}_{O_{2}\mathrm{peak}}$ with BFR, compared to without BFR, led to significantly greater reductions in tissue oxygen saturation at the VL and GM, and increased HR, ${\dot{V}}_{O_{2}}$, ${\dot{V}}_{CO_{2}}$, ${\dot{V}}_{E}$, BLA, RPE, discomfort and body instability. Participants rated BFR and non‐BFR jumping conditions as equally enjoyable. The acute physiological responses observed during BFR jumping in simulated lunar gravity align closely with those during low‐intensity BFR aerobic exercise on Earth, such as walking and cycling. Importantly, BFR aerobic exercise has been found to promote muscle hypertrophy and improve cardiovascular fitness (de Oliveira et al., 2016). Jumping with BFR, therefore, could serve as a practical, low resource exercise countermeasure within surface habitats during planetary exploration missions; it requires further studies to validate its effectiveness as a training intervention and countermeasure against disuse‐induced deconditioning.

### 4.2 Physiological and perceptual responses to blood flow restriction jumping in simulated lunar gravity

#### 4.2.1 Tissue oxygenation

Application of BFR at 60% LOP to the lower limbs during jumping in simulated lunar gravity resulted in a 2–3‐fold reduction in TOI at the VL compared to the ‘free‐flow’ non‐BFR control condition (∼20% vs. ∼8%). This reduction aligns closely with responses observed during BFR cycling on Earth, where tissue oxygenation progressively declines with increasing occlusion pressure due to attenuated blood flow (Kilgas et al., 2019; Kilgas et al., 2022; Lauver et al., 2022). Reducing blood flow, and thus oxygen delivery, to the exercising muscles was coupled with elevated metabolic stress, evidenced by increases in blood lactate, carbon dioxide output, minute ventilation and ratings of perceived exertion (Suga et al., 2010).

Deflation of the pneumatic tourniquet cuff during the 1‐min rest period rapidly returned VL TOI to baseline levels, a pattern consistent with terrestrial studies of TOI recovery via reactive hyperaemia during which an ∼10‐fold increase in vascular shear stress has been reported (Lavigne et al., 2024). This cyclical pattern of transient ischaemia followed by reperfusion is thought to stimulate angiogenesis and mitochondrial biogenesis, which are critical physiological adaptations shown to be impaired during disuse/spaceflight, highlighting the potential value offered by BFR in these contexts (Ferguson et al., 2018; Lavigne et al., 2024; Saatmann et al., 2021).

In contrast, responses in the GM were more variable on an individual level. During non‐BFR jumping, the GM TOI increased by ∼4–6%, whilst with BFR TOI remained close to resting baseline levels. Previous literature has observed that cycling with BFR induces a consistent decline in TOI within the GM by ∼6% compared to non‐BFR cycling (Corvino et al., 2017). In addition, performing ankle plantarflexions in a supine position with BFR has been found to significantly lower GM tissue oxygenation relative to without BFR (Yanagisawa and Sanomura, 2017). Though the average change in TOI of the GM seems to suggest that it was relatively unaffected, it is worthwhile highlighting the varied individual responses. Tissue oxygenation index of the GM in some individuals paralleled that of the VL (i.e., a rapid decline upon jumping followed by a plateau), whereas other participants displayed a decline and partial TOI restoration. This latter response has been observed during dynamic plantarflexion exercise and coincides with an increase in total haemoglobin and femoral arterial blood flow, suggesting that a partial rise in GM TOI during exercise may be attributed to increases in local blood flow to the muscle (Quaresima et al., 2001). An unexpected finding was that some participants also displayed an elevated GM TOI during jumping. Muscle oxygenation can indeed increase during exercise, but typically within non‐exercising muscle groups (e.g., forearms during cycling) as a result of an increase in blood flow (Nagasawa, 2008). Though blood flow distal to the site of BFR is attenuated with the degree of occlusion, it can still be elevated above resting levels during exercise, thereby providing a possible explanation for unchanged or elevated GM TOI during BFR and non‐BFR jumping conditions (Kilgas et al., 2019). Such an interpretation leads to an interesting contemplation that some individuals may not naturally utilise the GM, and perhaps the calf more generally, when jumping in simulated lunar gravity. This is an important consideration as the calf is one of, if not the, most susceptible muscle groups to deconditioning within disuse or microgravity environments (Comfort et al., 2021; LeBlanc et al., 1995; Scott et al., 2023; Winnard et al., 2019).

#### 4.2.2 Cardiorespiratory and metabolic responses

Jumping with BFR elicited a modest augmentation of the cardiovascular response. Heart rate increased by 10–23 beats min^−1^ compared with BFR, relative to non‐BFR conditions. This mirrors findings from terrestrial BFR walking and cycling studies, whereby the reduction in venous return and elevated metabolic stress results in a compensatory sympathetic activation to increase heart rate (Karabulut and Garcia, 2017; Kilgas et al., 2022). The impact of BFR on oxygen consumption was relatively small, with changes remaining ≤3 mL kg^−1^ min^−1^ (equivalent to ≤5% ${\dot{V}}_{O_{2}\mathrm{peak}}$), which has been reported in other studies examining low‐intensity BFR aerobic exercise (Corvino et al., 2017; Kilgas et al., 2022). Significant increases in ${\dot{V}}_{CO_{2}}$ were observed during BFR versus non‐BFR jumping; however, they were again relatively modest at ≤4 mL kg^−1^ min^−1^ (equivalent to ≤7% ${\dot{V}}_{CO_{2}}$_max_). Blood lactate concentrations were unaffected during non‐BFR jumping and remained near resting values; however, with the addition of BFR, BLA increased up to an average of 3–4 mmol L^−1^, which is of a similar magnitude to previous findings examining low‐intensity BFR aerobic exercise (∼3–5 mmol L^−1^) (Corvino et al., 2017; Kilgas et al., 2022). The accumulation of metabolites during exercise is recognised as an important stimulus for protein signalling and/or satellite cell proliferation to induce muscle growth via proposed mechanisms including increased recruitment of fast‐twitch fibres, muscle damage, inflammatory processes associated with remodelling, and production of reactive oxygen species (Pearson and Hussain, 2015). Significant increases in minute ventilation were observed by 7–16 L min^−1^ (equivalent to ∼5–10% ${\dot{V}}_{\mathrm{Emax}}$), primarily driven by an increase in tidal volume due to non‐significant changes in breathing frequency. This response is secondary to elevated ${\dot{V}}_{CO_{2}}$ and H^+^, both of which increase respiratory drive. Previous research has similarly observed elevated ${\dot{V}}_{CO_{2}}$ and ${\dot{V}}_{E}$ during BFR aerobic exercise, reflecting an amplified anaerobic contribution to energy production (Corvino et al., 2017; Kilgas et al., 2022; Pugh et al., 2024). Together, these findings indicate that supplementing low intensity jumping in simulated lunar gravity with BFR can effectively augment metabolic and cardiovascular stress in a similar manner as seen in terrestrial settings.

#### 4.2.3 Mechanical loading and fatigue

On Earth (i.e., 100% bodyweight), jumping activities can impose vGRFs of ∼400–500% bodyweight, with cyclical high‐magnitude loading cycles recognised as a key stimulus for improving bone mineral density, tendon stiffness, muscle morphology, jump performance and lower limb strength (McNair and Prapavessis, 1999; Ramírez‐delaCruz et al., 2022; Wallace et al., 2010). By contrast, BFR training is typically performed with low external loads equivalent to 20–40% 1‐repetition maximum or <50% ${\dot{V}}_{O_{2}\mathrm{peak}}$ to facilitate use in load‐comprised populations (e.g., post‐anterior cruciate ligament surgery) and to prevent premature fatigue due to reduced blood supply to contracting skeletal muscle. In our present work, low‐intensity jumping in simulated lunar gravity, at a jump height equivalent to 40% ${\dot{V}}_{O_{2}\mathrm{peak}}$, resulted in peak vGRFs of ∼70–90% bodyweight. Differences between BFR and non‐BFR jumping conditions were relatively minor (∼10% bodyweight). Though jumping at low intensities with average peak impact forces <100% bodyweight could be viewed as having a diminished potential for improving/protecting bone health, BFR induces local tissue hypoxia and augments interstitial fluid shear‐stress, both of which can activate key signalling pathways involved in bone adaptation (Hughes and Centner, 2024). Further research is required to establish the long‐term effects of BFR training on bone structure and function, for this may offer a unique low‐load exercise modality for bone health with relevance to astronaut populations.

Acute reductions in neuromuscular function have been reported following a single BFR training session, attributed to both peripheral (e.g., ionic disturbances) and central (e.g., reduced motor output) mechanisms (Kilgas et al., 2022; Reis et al., 2019). The present study identified a significant effect of time for the pre–post peak knee extension force assessment across conditions; however, the lack of a significant effect of condition indicated that the magnitude of change was not different between BFR and non‐BFR jumping conditions. Low‐intensity BFR cycling at 60% and 80% LOP has been found to induce significant reductions in post‐exercise knee extension MVIC torque by ∼18% and ∼40%, respectively and also voluntary activation by ~23% (BFR at 80% LOP condition only), providing evidence of BFR‐induced peripheral and central fatigue (Kilgas et al., 2022). Additionally, Reis et al. reported reductions in peak torque following BFR exercise, but only when applied at 80% LOP (∼6%), whereas no changes in peak torque were evident when using BFR at 40% and 60% LOP (Reis et al., 2019). These findings provide evidence of neuromuscular fatigue with BFR aerobic exercise, particularly when using higher relative occlusion pressures. In addition, the present study employed intermittent BFR (i.e., the pneumatic tourniquet cuff was deflated during each rest period), which has been shown to attenuate post‐exercise losses in maximal force production (Corvino et al., 2022). Our findings suggest that jumping with intermittent BFR at 60% LOP can effectively augment metabolic stress with relatively modest post‐exercise fatigue, which from an operational perspective could help reduce the impact of exercise‐induced fatigue on subsequent physically demanding work (e.g., EVAs) or timelines separating these activities.

#### 4.2.4 Perceptual responses and safety

Participants reported significantly higher RPE and discomfort during BFR jumping. During the non‐BFR jumping condition, RPE and discomfort were rated as ‘very light’ (9/20) and ‘very weak’ (1/11), respectively. During BFR jumping, RPE and discomfort increased to mean ratings of ‘very hard’ (RPE 17/20) and ‘strong’ to ‘very strong’ (6‐7/11), respectively, but with notable individual variability. These subjective responses are consistent with the known sensations associated with BFR exercise (Bielitzki et al., 2024; Spitz et al., 2022). Despite greater RPE and discomfort, participants rated BFR jumping as equally enjoyable to non‐BFR jumping, indicating that the elevated perceptual demands did not diminish the acceptability of exercise, supporting the use of BFR jumping as part of an exercise countermeasure programme. Whilst other studies have observed reductions in exercise enjoyment following a bout of BFR versus non‐BFR exercise, there are important factors to consider with regard to how enjoyment may be affected by the BFR prescription used. The present study employed intermittent BFR (inflation–deflation cycles), allowing for a period of reperfusion, which has been shown to reduce pain compared to continuous BFR (Fitschen et al., 2014). In addition, BFR was applied as a percentage of LOP, accounting for individual differences in the amount of pressure required to induce full arterial occlusion; failure to do so results in an unknown degree of arterial occlusion (McEwen et al., 2019). The tourniquet cuff used was also contoured to the shape of the limb to improve pressure distribution around the circumference of the limb. Studies reporting reductions in enjoyment with BFR have used continuous BFR during walking (15 min) or resistance exercise (30–15–15–15 repetition scheme) and applied using a fixed arbitrary pressure of 200 mmHg using an unspecified tourniquet cuff (Mok et al., 2020; Suga et al., 2021). One factor that cannot be dismissed, however, is whether the novelty of being suspended in simulated lunar gravity impacted exercise enjoyment, pointing toward the need for a future training study in which it would be possible to understand the effect of repeated BFR jumping sessions on exercise adherence‐related parameters.

Participants reported increased instability during BFR jumping, which is a matter that should be examined further, as the design of the suspension mechanism employed in the study helped minimise fall risk. Use of a hypogravity analogue with increased freedom‐of‐movement (e.g., vertical bodyweight suspension) can inform whether instability during jumping (with and without BFR) may pose a risk to the user (e.g., falls or adverse joint loads during take‐off/landing) that require mitigation (e.g., habitat structures/beams to correct body position/trajectory and facilitate a safe jumping technique).

#### 4.2.5 Implications for exercise in lunar habitats

The present study demonstrates that it is possible to perform low‐intensity bodyweight jumping at 40% ${\dot{V}}_{O_{2}\mathrm{peak}}$ in simulated lunar gravity with intermittent lower limb BFR, outlining a promising way that bodyweight jumping could be integrated into lunar exercise countermeasure programmes. The physiological and perceptual responses induced by BFR jumping, including reduced muscle oxygenation, elevated heart rate, gas exchange, ventilation, blood lactate, perceived exertion and discomfort, closely mirror findings from terrestrial BFR aerobic exercise (Corvino et al., 2017; Kilgas et al., 2022). This is supported by recent evidence demonstrating that continuous bodyweight jumping in simulated lunar gravity can be used as a form of cardiovascular exercise (Swain et al., 2025b). These parallels may imply that BFR jumping in hypogravity can elicit systemic stressors comparable to Earth‐based BFR aerobic exercise protocols, which have been shown to improve both aerobic capacity and muscle size and strength (de Oliveira et al., 2016). Furthermore, BFR may activate signalling pathways associated with osteogenesis (via hypoxia‐inducible factors, oxidative stress and hormonal cascades), potentially addressing bone loss that is a major concern during periods of reduced mechanical loading (e.g., long‐duration spaceflight) (Hughes and Centner, 2024). Future research should explore the long‐term training effects of BFR jumping in low‐gravity settings, including in other levels of hypogravity (e.g., Martian gravity [0.38 *g*]), in a disuse analogue (e.g., bedrest or dry immersion) to establish how well this form of exercise can mitigate multi‐system deconditioning, a critical endeavour to informing the use of BFR jumping as a countermeasure in future planetary exploration missions.

Jumping with BFR offers several potential benefits when considered as a potential exercise countermeasure in hypogravity environments. Little equipment is required, other than a lightweight BFR device. Habitat headroom restrictions may limit the use of high jump heights, which appear necessary for augmenting metabolic rates to desired cardiovascular training intensities (Swain et al., 2025b). Jumping with BFR increases the feasibility of bodyweight jumping as a potential exercise countermeasure within the potential height constraints of lunar habitats. Combined aerobic and muscular adaptations could also help reduce exercise timeframes and potentially preclude or reduce requirements for standalone ‘aerobic’ and ‘resistive’ exercise. Use of low intensities that, by definition, reduces the metabolic cost of exercise, coupled with short exercise sessions (∼10–20 min), would reduce exercise‐related burdens on environmental control and life support systems including O_2_ consumption, CO_2_, heat and moisture production, and overall energy expenditure, relative to high‐intensity exercise (Kilgas et al., 2022; Laws et al., 2020). In addition, low‐load exercise could reduce the risk of strain‐related injuries and remains a valuable exercise intervention in the scenario of a musculoskeletal injury during mission, given the recognised benefits of BFR in clinical rehabilitation settings (Cognetti et al., 2022; Hughes et al., 2017). This seems particularly relevant as astronauts are likely at an increased risk of musculoskeletal injury during surface EVAs that could be performed up to ∼24 h a week (NASA, 2020; NASA, 2022a). These factors collectively underscore the potential relevance of BFR jumping in lunar surface habitats, particularly where logistical, spatial and hardware constraints are expected.

### 4.3 Limitations

The present study was performed in standard laboratory atmospheric conditions equivalent to sea level on Earth (∼14.7 psi and 21% O_2_). Lunar and Martian surface habitats are, however, expected to operate at hypobaric hypoxia (8.2 psi and 32% O_2_) to reduce decompression lengths and risk of decompression sickness, to facilitate frequent surface EVAs (Norcross et al., 2015). Previous research shows that performing BFR exercise in combination with systemic hypoxia amplifies local muscle tissue deoxygenation without compromising performance, suggesting that systemic hypoxia might amplify the acute physiological stress provided by BFR during low external‐intensity exercise without compromising training workload (Wang et al., 2023). It would be valuable to characterise BFR training adaptations when performed in the expected surface habitat atmosphere (hypobaric hypoxia) versus normoxia.

The jumping exercise intensity was prescribed at 40% ${\dot{V}}_{O_{2}\mathrm{peak}}$, but the ${\dot{V}}_{O_{2}\mathrm{peak}}$ test was performed using cycle ergometry in an upright (1 *G*_z_) posture. It is known that ${\dot{V}}_{O_{2}\mathrm{peak}}$ can be dependent on the exercise modality in which it is derived; for instance, ${\dot{V}}_{O_{2}\mathrm{peak}}$ is ∼5–10% lower during cycling compared to running (Hermansen and Saltin, 1969). Body posture also significantly affects ${\dot{V}}_{O_{2}\mathrm{peak}}$, which can be up to ∼20% lower in a supine, relative to upright, position during cycle ergometry (Dillon et al., 2021). It must therefore be taken into consideration that the jumping exercise intensity (%${\dot{V}}_{O_{2}\mathrm{peak}}$) is expressed relative to peak metabolic rates during upright cycle ergometry. Due to the postural influence on ${\dot{V}}_{O_{2}\mathrm{peak}}$, it is likely that the relative jumping exercise intensity would be higher if ${\dot{V}}_{O_{2}\mathrm{peak}}$ had been determined in the 9.5° head‐up tilt position. It is, however, unclear how different an individual's ${\dot{V}}_{O_{2}\mathrm{peak}}$ would be when performed between cycling or jumping (at ∼20% bodyweight) modalities, nor whether it is possible to elicit ${\dot{V}}_{O_{2}\mathrm{peak}}$ whilst jumping in lunar gravity. The present study also used a maximal voluntary isometric knee extension test as a simple metric for post‐exercise fatigue. This could be improved further by exploring neuromuscular fatigue of both central and peripheral origins.

The VGSS provided a simulated lunar gravity environment through whole body suspension at 9.5° head‐up tilt. Suspending the body via fixed overhanging ropes results in the body conforming to the path of a pendulum during positive or negative displacement from the default standing position. The component of the pendulum acting along the longitudinal axis of the body, therefore, causes deceleration and acceleration during the ascending and descending phases of the jump, respectively. As the body moves away from the supporting surface under the feet (e.g. during a jump), the suspension rope inclination angle will increase above the 9.5° starting inclination, thus increasing the axial gravitational acceleration acting on the participant above the target hypogravity level due to the pendulum effect (Swain et al., 2025c). This deviation (increase) of the axial gravitational acceleration will lead to lower jump heights and higher jumping frequencies than would be expected in actual lunar gravity. Use of low jump heights would also have also reduced the influence of the pendulum effect. This may, however, have resulted in a progressive overestimation of the energetic cost of jumping during the incremental jumping test that was required to prescribe the exercise intensity. Due to the design of the VGSS, frontal motion, whilst possible, is limited due to the suspension ropes, wanting to return to equilibrium following any lateral rope tilt. This leads to much greater stability in the medio‐lateral direction during jumping tasks than would otherwise be present in actual lunar gravity. A key question remains as to whether there are any challenges to jumping continuously in hypogravity with complete freedom‐of‐movement.

### 4.4 Conclusion

Low‐intensity (40% ${\dot{V}}_{O_{2}\mathrm{peak}}$) bodyweight jumping with BFR in simulated lunar gravity amplifies the metabolic, cardiovascular and perceptual responses to exercise compared to without BFR, and generates peak vGRFs between ∼70% and 90% bodyweight. The acute physiological and perceptual responses observed during BFR jumping closely mirror those observed during BFR walking and cycling on Earth that have been found to elicit positive muscular and cardiovascular training adaptations. Our findings highlight a unique exercise modality (bodyweight jumping with BFR) that could be integrated into surface exercise countermeasure programmes where logistical, spatial and hardware constraints are expected. Further long‐term studies are required to document the effects of BFR jumping in hypogravity settings as a chronic training intervention and countermeasure against disuse‐induced deconditioning.

## AUTHOR CONTRIBUTIONS

The study was conducted in the Aerospace Medicine & Rehabilitation Laboratory (Northumbria University, UK). All authors contributed to (1) the conception or design of the work or (2) the acquisition, analysis or interpretation of data for the work and (3) drafting the work or revising it critically for important intellectual content. All authors have read and approved the final version of this manuscript and agree to be accountable for all aspects of the work in ensuring that questions related to the accuracy or integrity of any part of the work are appropriately investigated and resolved. All persons designated as authors qualify for authorship, and all those who qualify for authorship are listed.

## CONFLICT OF INTEREST

Luke Hughes serves in a scientific and educational capacity for the manufacturer of the blood flow restriction device used in the present study. The other authors declare no conflicts of interest.

## FUNDING INFORMATION

None.

## Supporting information



Table. S1. Details of the BFR device and procedures.

Table S2. Statistical analysis summary report.

Video S1. A user jumping in simulated lunar gravity within the VGSS.

## Data Availability

Source data for this study are openly available at https://doi.org/10.5281/zenodo.17879075.
