# Supplementary material for: Jumping in simulated lunar gravity with blood flow restriction as a potential exercise countermeasure: The acute physiological effects
Source: Exp Physiol. 2026 Jun 17;111(8):3699–717. doi: 10.1113/EP093652 (PMC13394901; doi:10.1113/EP093652)
Supplement: Supplementary file 1 — Table. S1. Details of the BFR device and procedures. [file EPH-111-3699-s001.docx]

Supplementary Table S1. Blood Flow Restriction (BFR) Device Properties

| BFR Instrument Apparatus Capabilities | |
| --- | --- |
| Manufacturer and model | Delfi Medical – Personalized Tourniquet System for BFR |
| Method of pressure measurement | Automatic LOP measurement via cuff |
| Pressure regulation | 20 – 350 mmHg  ± 6 mmHg of set-point (10-second average under non-transient conditions without external leaks) |
| Validity and reliability of limb occlusion pressure measurement | **References:**   - Masri, B.A., Day, B., Younger, A.S. and Jeyasurya, J., 2016. Technique for measuring limb occlusion pressure that facilitates personalized tourniquet systems: a randomized trial. *Journal of Medical and Biological Engineering*, *36*, pp.644-650. - Hughes, L., McEwen, J. 2021. Investigation of clinically acceptable agreement between two methods of automatic measurement of limb occlusion pressure: a randomized trial. BMC Biomedical Engineering, 3, pp. 1-8. - Hughes, L., Jeffries, O., Waldron, M., Rosenblatt, B., Gissane, C., Paton, B. and Patterson, S.D., 2018. Influence and reliability of lower-limb arterial occlusion pressure at different body positions. *PeerJ*, *6*, p.e4697. - McEwen USRE50013 Tourniquet system for personalized restriction of blood flow - McEwen EP3171794 Tourniquet system for personalized restriction of blood flow |
| Tourniquet Cuff Properties | |
| Manufacturer and model | Delfi Medical Easi-Fit BFR Cuff and Matching Limb Protection Sleeve |
| Cuff selection | Limb size chart by manufacturer |
| Cuff width (width x length) | 24” x 4.5”  34” x 4.5” |
| Material | Nylon |
| Type of internal bladder system | Single bladder system -  Fully encircles the limb |
| Cuff shape | Contoured |
| Internal bladder length | 24 – 21.5”  34 - 31.5” |
| BFR Pressure Prescription | |
| Limb occlusion pressure (mmHg)  (n = 14) | 200 ± 18 mmHg (left leg)  194 ± 15 mmHg (right leg) |
| Posture used for measurement of limb occlusion pressure | LOP can be significantly influenced by body posture and body orientation, therefore, was measured with the participant standing quietly in simulated Lunar gravity (i.e., 9.5° head-up tilt bodyweight suspension) immediately prior to commencing the BFR jumping exercise.   - Hughes, L., Jeffries, O., Waldron, M., Rosenblatt, B., Gissane, C., Paton, B. and Patterson, S.D., 2018. Influence and reliability of lower-limb arterial occlusion pressure at different body positions. *PeerJ*, *6*, p.e4697. - Swain, P., Caplan, N. and Hughes, L., 2024. Blood flow restriction: The acute effects of body tilting and reduced gravity analogues on limb occlusion pressure. *Experimental Physiology*. |
| Timings and pressure application | Inflated to 60% LOP ~5-seconds prior to jumping and maintained throughout each of the six jumping sets (2-minute) and deflated completely during the rest periods (1-minute). |
| Target vs actual pressure applied | Tourniquet cuff pressure was not directly measured in the present study. The Delfi Personalized Tourniquet System for BFR has been shown to maintain pressure within approximately ±10% of the prescribed LOP during lower limb exercise.   - Swain, P., McEwen, J., Lai, T. and Hughes, L., 2025. Tourniquet cuff pressure during blood flow restriction exercise. *Frontiers in Sports and Active Living*, *7*, p.1582387. |

*Abbreviations: LOP, limb occlusion pressure.*
