# Supplementary material for: Jumping in simulated lunar gravity with blood flow restriction as a potential exercise countermeasure: The acute physiological effects
Source: Exp Physiol. 2026 Jun 17;111(8):3699–717. doi: 10.1113/EP093652 (PMC13394901; doi:10.1113/EP093652)
Supplement: Supplementary file 2 — Table S2. Statistical analysis summary report. [file EPH-111-3699-s002.docx]

Supplementary Table S2. Statistical Analysis Summary Report

| Measure | Timepoint(s) | Exclusion | Transformation | Reason | Change in Statistical Significance/Interpretation | Manuscript Reporting |
| --- | --- | --- | --- | --- | --- | --- |
| Jump Height | All exercise sets (x6) | n = 1 | Log10 | *Outlier.* One participant had an exceptional VO_2max_ (73ml/kg/min) resulting in the jump height at 40% VO_2max_ being far greater than the cohort average (54cm vs. 35 ± 4cm) causing the jump height probability distribution to be skewed and kurtotic.  Distribution of data in some sets were still slightly skewed following outlier removal, thus, Log10 transformation was explored. | No change following outlier removal or data transformation. | Original analysis without data exclusion/transformation. |
| Jump frequency | All exercise sets (x6) | None | None | NA | NA | NA |
| Jump depth | All exercise sets (x6) | None | None | NA | NA | NA |
| Peak vertical ground reaction force | All exercise sets (x6) | None | None | NA | NA | NA |
| Vastus lateralis ∆TOI | All exercise sets (x6) and rests (x5) | None | None | NA | NA | NA |
| Gastrocnemius medialis ∆TOI | All exercise sets (x6) and rests (x5) | None | None | NA | NA | NA |
| VO_2_ | All exercise sets (x6) and rests (x5) | n=1 | None | *Outlier.* One participant with an exceptional VO_2max_ (73ml/kg/min) also had high VO_2_ values during the jumping sets causing the probability distributions to be skewed and kurtotic. | No change in main effects. Two simple pairwise comparisons became non-significant after outlier removal (Jump Set 4 Rest and Jump Set 5 Exercise). | Two-way ANOVA performed with outlier removal. |
| VCO_2_ | All exercise sets (x6) and rests (x5) | n = 1 | None | Same justification as VO_2_ | No change in main effects or pairwise comparisons. | Two-way ANOVA performed with outlier removal. |
| VE | All exercise sets (x6) and rests (x5) | None | None | NA | NA | NA |
| BF | All exercise sets (x6) and rests (x5) | None | None | NA | NA | NA |
| HR | All exercise sets (x6) and rests (x5) | None | None | NA | NA | NA |
| Blood lactate | All exercise sets (x6) | n = 3 | None | Three potential outliers removed as identified in box plots. | No change. | Original analysis without data exclusion. |
| Peak Knee Extension Force | Pre-post | None | None | NA | NA | NA |
| Rating of perceived exertion | All exercise sets (x6) | None | None | NA | NA | NA |
| Discomfort | All exercise sets (x6) | None | None | NA | NA | NA |
| Movement instability | All exercise sets (x6) | None | None | NA | NA | NA |
| Physical activity enjoyment | Post-exercise | None | None | NA | NA | NA |

Abbreviations: TOI, tissue oxygenation index.
